# A novel approach to guide GD2-targeted therapy in pediatric tumors by PET and [^64^Cu]Cu-NOTA-ch14.18/CHO

**DOI:** 10.7150/thno.92481

**Published:** 2024-01-20

**Authors:** Nils Florian Trautwein, Johannes Schwenck, Christian Seitz, Ferdinand Seith, Eduardo Calderón, Sebastian von Beschwitz, Stephan Singer, Gerald Reischl, Rupert Handgretinger, Jürgen Schäfer, Peter Lang, Bernd J. Pichler, Johannes H. Schulte, Christian la Fougère, Helmut Dittmann

**Affiliations:** 1 Department of Nuclear Medicine and Clinical Molecular Imaging, University of Tübingen.; 2 Werner Siemens Imaging Center, Department of Preclinical Imaging and Radiopharmacy, University of Tübingen.; 3 Cluster of Excellence iFIT (EXC 2180) "Image-Guided and Functionally Instructed Tumor Therapies", University of Tübingen.; 4 Department of Pediatric Hematology and Oncology, University of Tübingen.; 5 Department of Diagnostic and Interventional Radiology, University of Tübingen.; 6 Department of Pathology, Eberhard Karls University Tübingen, 72076 Tübingen, Germany.; 7 German Cancer Consortium (DKTK), Partner Site Tübingen, Germany.

## Abstract

**Background:** The tumor-associated disialoganglioside GD2 is a *bona fide* immunotherapy target in neuroblastoma and other childhood tumors, including Ewing sarcoma and osteosarcoma. GD2-targeting antibodies proved to be effective in neuroblastoma and GD2-targeting chimeric antigen receptors (CAR)- expressing T cells as well as natural killer T cells (NKTs) are emerging. However, assessment of intra- and intertumoral heterogeneity has been complicated by ineffective immunohistochemistry as well as sampling bias in disseminated disease. Therefore, a non-invasive approach for the assessment and visualization of GD2 expression *in-vivo* is of upmost interest and might enable a more appropriate treatment stratification.

**Methods:** Recently, [^64^Cu]Cu-NOTA-ch14.18/CHO (^64^Cu-GD2), a radiolabeled GD2-antibody for imaging with Positron-Emission-Tomography (PET) was developed. We here report our first clinical patients' series (n = 11) in different pediatric tumors assessed with ^64^Cu-GD2 PET/MRI. GD2-expression in tumors and tissue uptake in organs was evaluated by semiquantitative measurements of standardized uptake values (SUV) with PET/MRI on day 1 p.i. (n = 11) as well as on day 2 p.i. (n = 6).

**Results:** In 8 of 9 patients with suspicious tumor lesions on PET/MRI at least one metastasis showed an increased ^64^Cu-GD2 uptake and a high tracer uptake (SUV_max_ > 10) was measured in 4 of those 8 patients. Of note, sufficient image quality with high tumor to background contrast was readily achieved on day 1. In case of ^64^Cu-GD2-positive lesions, an excellent tumor to background ratio (at least 6:1) was observed in bones, muscles or lungs, while lower tumor to background contrast was seen in the spleen, liver and kidneys. Furthermore, we demonstrated extensive tumor heterogeneity between patients as well as among different metastatic sites in individual patients. Dosimetry assessment revealed a whole-body dose of only 0.03 mGy/MBq (range 0.02-0.04).

**Conclusion:** ^64^Cu-GD2 PET/MRI enables the non-invasive assessment of individual heterogeneity of GD2 expression, which challenges our current clinical practice of patient selection, stratification and immunotherapy application scheme for treatment with anti-GD2 directed therapies.

## Introduction

Although the overall survival of children suffering from all types of cancer is reaching 80%, the treatment of metastatic disease has not significantly improved in some entities like sarcomas in recent years 1.

The disialoganglioside GD2 is a tumor-associated antigen, which is overexpressed on cell surfaces of various cancer entities 2-4. Because of its restricted expression in physiological tissues GD2 is a reasonable target for cancer immunotherapy 5 and various GD2-targeting antibodies have been applied in different cancer entities such as melanoma, neuroblastoma and osteosarcoma 6-9. Immunotherapy with ch14.18 (dinutuximab) a chimeric monoclonal antibody against GD2, revealed a significant benefit in addition to standard therapy with retinoids improving overall survival in patients with high-risk neuroblastoma 10 and consecutively obtained a registered indication for maintenance therapy by the U.S. Food and Drug Administration (FDA) 11. Recently, ch14.18/CHO (dinutuximab beta) an antibody produced in chinese hamster ovarian (CHO) cells was approved by the European Medicines Agency (EMA) in 2017 12 and became standard of care for patients with high-risk neuroblastoma who have achieved at least a partial response to previous multimodal treatment 13. However, this treatment can be associated with several adverse events, such as capillary leak syndrome, neuropathic pain and fever 14. Though some side effects can be reduced by premedication and a decreased infusion rate, dinutuximab beta handling remains a clinical challenge in pediatric oncology 15 and its use is not yet established in other GD2-positive tumor entities. Clinical trials are currently underway evaluating the treatment of osteosarcoma (NCT02484443), leiomyosarcoma (NCT05080790) and initial trials in Ewing's sarcoma are ongoing 16.

Recently, GD2-targeting chimeric antigen receptor (CAR) modified natural killer T cells (NKT) as well as T cell therapy 17. In particular the CAR T cell approach has shown promising antitumorigenic effects in the treatment of relapsed or refractory high-risk neuroblastoma with a response rate of 63% 18. In this study GD2 antigen expression as assessed by flow cytometric analysis of bone marrow samples before treatment or after treatment failure was shown to be positive in all patients, even in those with progressive disease. Thus, the authors concluded that mechanisms of resistance in neuroblastoma other than antigen loss might be responsible 18. However, although GD2 expression is considered to be nearly ubiquitous in neuroblastoma, a complete or partial lack of its expression has been described in up to 12% of cases 19. In addition, a higher intra- and interindividual variability in GD2 overexpression was shown in osteosarcoma and Ewing's sarcoma ranging from 40 to 100% of the cases 20, 21. Currently, expression analysis of GD2 is mainly performed *in-vitro* using immunohistochemistry, which requires individual tumor samples and is therefore susceptible to sampling bias. In addition, this procedure cannot provide a holistic representation of GD2 expression and thus cannot reveal potential intraindividual heterogeneity. Consequently, a non-invasive diagnostic marker that enables both visualization and quantification of GD2 expression *in-vivo* is of utmost interest for appropriate treatment stratification, especially in those cancers where variable GD2 expression may occur. This approach would have the potential to expand the use of GD2-targeting immunotherapies in cancers other than neuroblastoma, such as sarcoma, glioma or triple negative breast cancer, but also to prevent ineffective therapies in tumors with poor GD2-expression and to avoid the possibly severe side effects 18. Furthermore, PET imaging with a radiolabeled GD2 antibody might provide a deeper insight into tissue penetration of GD2 antibody *in-vivo* revealing restricted access to the target and thus enable an early prediction of treatment failure*.*

In response to this need, non-invasive GD2 imaging has been extensively studied in preclinical settings by several investigators 22-24. Recently, our group has developed the PET tracer [^64^Cu]Cu-NOTA-ch14.18/CHO (^64^Cu-GD2), which enabled to detect GD2 expression in a preclinical setting and initially showed to be clinically applicable in neuroblastoma as well as osteosarcoma 25, 26.

We here report the first multi-patient cohort of tumor patients studied with ^64^Cu-GD2 PET/MRI. Our goal was to evaluate intra- and interindividual GD2 expression for tumor targeting and to quantitatively reveal ^64^Cu-GD2 biodistribution. In addition, important specifically in children and young adults, we aimed to approximate the irradiation exposure of ^64^Cu-GD2 in a subset of patients undergoing dual timepoint PET/MRI examination.

## Material and Methods

### Patients

A total of 11 patients (mean age: 13.9 ± 5.0 years) underwent ^64^Cu-GD2 PET/MRI. All patients had histologically confirmed disease (6 patients suffered from neuroblastoma, 3 patients had osteosarcoma and 2 patients had Ewing's sarcoma). Patient characteristics are provided in Table 1. 4 of them were scanned because of suspected cancer recurrence and the remaining 7 patients for the evaluation of GD2 expression in already known tumor lesions.

All patients and/or their parents gave written informed consent to undergo ^64^Cu-GD2 PET/MRI following the regulations of the German Pharmaceuticals Act (“Arzneimittelgesetz” AMG §13(2b)). All individuals were facing an unmet diagnostic challenge that could not be solved with standard diagnostic, either insufficient tumor delineation before planned surgery/ external radiation or need to evaluate the GD2 expression before administration of dinutuximab beta. This retrospective analysis was approved by the institutional review board (decision 030/2023BO2). The need for written informed consent was waived for this study.

### Adverse Events

Standard vital parameters were documented and patients were asked to report any abnormalities during or after ^64^Cu-GD2 infusion.

### Radiopharmaceuticals

Dinutuximab beta was provided by EUSA-pharma. Synthesis and labeling of ^64^Cu-GD2 was performed as previously described 25. In short, 1 mg of NOTA-conjugated antibody was incubated with a buffered solution of 500 MBq of ^64^Cu resulting in approximately 0.08 to 0.47 mg of dinutuximab beta injected.

### PET/MRI Data Acquisition

To avoid adverse reactions to the antibody, H1 and H2 receptor antagonists were given intravenously at least 30 min prior to tracer injection. ^64^Cu-GD2 was administered i.v. over 30 min using a dosage of 2-3 MBq per kg/BW in the first four patients and was afterwards adapted to 4-5 MBq per kg/BW to improve the count rate (details provided in table 1). A first simultaneous PET/MRI examination (day 1) was performed 21 h p.i. (range 17-25 h) using a whole-body PET/MRI system (Biograph mMR®, Siemens Healthineers, Germany). In six patients a second PET/MRI scan (day 2) was executed at 43 h p.i. (range: 41-44 h). PET images (6 min acquisition per bed position, 4-6 bed positions) were reconstructed using an OSEM-3D algorithm and corrected for scatter and attenuation using an MR-based segmentation as described previously 27, 28. Simultaneous to PET imaging, a coronal T1-weighted 3D-encoded spoiled gradient-echo sequence with double-echo for Dixon-based fat-water separation for attenuation correction, a coronal T2 turbo inversion recovery magnitude (TIRM), a coronal T2 short inversion time inversion recovery (STIR), a transaxial T2 half-Fourier-aquired single shot turbo spin echo (HASTE) and transverse diffusion-weighted (DWI) echo-planar imaging sequences were acquired.

### Semiquantitative Analysis of ^64^Cu-GD2 uptake

^64^Cu-GD2 uptake measured with PET was quantified by means of VOI based mean and maximum standardized uptake values (SUV_mean_ and SUV_max_) using Hermes Affinity Viewer® software (Hermes Medical Solutions, Stockholm, Sweden). To quantify ^64^Cu-GD2 uptake in tumor lesions, a relative 41% of maximum iso-contour VOI was used. The normal tissue uptake was evaluated with a 1 ml spheric VOI for small organs (thyroid, parotid gland, left ventricle myocardium, bloodpool in aorta descendens and pancreas) and by a 3 ml spheric VOI for larger organs (brain, lung, liver, muscle, spleen, kidneys and in a non-metastatic lumbar vertebra for the bone marrow). Tumor to background ratio (TBR_mean_) was assessed for various areas and organs.

### Visual Analysis of ^64^Cu-GD2 uptake

A four-point Likert scale was defined for the visual assessment of ^64^Cu-GD2 uptake in the tumor areas compared to various background regions.

Likert 0: ^64^Cu-GD2 < bone marrow

Likert 1: ^64^Cu-GD2 uptake ≥ bone marrow; < bloodpool

Likert 2: ^64^Cu-GD2 uptake ≥ bloodpool

Likert 3: ^64^Cu-GD2uptake >> bloodpool

GD2 positive lesions were defined as Likert 2 or 3.

### Image quality

The image quality was subjectively evaluated by the readers. In addition, a coefficient of variation (CoV) was determined for objective analysis. This coefficient is considered a measure of the statistical technical variation in PET imaging 29. For this purpose, a 14 ml spherical volume of interest (VOI) was placed in the right liver lobe and the standardized uptake value (SUV) was measured. CoV was calculated using the following formula:



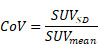



### Biodistribution and Radiation Dosimetry

Approximated dosimetry was calculated in the six patients scanned on two consecutive days. Radiation absorbed doses in organs such as liver, kidneys, lung or spleen and in tumor lesions were estimated from the ^64^Cu time-integrated activity coefficient (TIAC) in the predefined regions of interest. Radioactivity concentration was estimated from VOIs defined from MRI images at each timepoint using Hermes Affinity Viewer® software. OLINDA® software (Vanderbilt University, USA) was used for dosimetric analysis throughout.

### Histology and Immunohistochemistry

Formalin-fixed and paraffin-embedded tissue sections were stained with hematoxylin and eosin (H&E). Immunohistochemistry was performed on a Ventana BenchMark ULTRA instrument using the Snp88 clone for synaptophysin (DCS-Diagnostics, Hamburg, Germany), the DAK-A3 clone for Chromogranin A (DAKO Products Agilent, Santa Clara CA, USA) and the 14.G2a clone for GD2 (Merck Millipore, Burlington MA, USA).

### Statistical Analysis

Normality of distribution was tested using the Shapiro-Wilk test. Mann-Whitney U-Test was performed for not normally distributed variables. The statistical analysis was performed using GraphPad Prism® 9.4 software (GraphPad Software, Boston MA, USA). p < 0.05 was considered as significant.

## Results

### Tumor detection in ^64^Cu-GD2 PET/MRI and safety

In 2 of 4 patients with suspected cancer recurrence, the presence of malignant tumor lesions could be excluded by PET/MRI, since no suspicious lesions were detectable (one patient with neuroblastoma and one patient with Ewing's sarcoma). Consequently, PET/MRI imaging detected metastatic tumor disease, in 9 of 11 patients (Table 1). Of these, 5 showed bone, 3 lung, 2 lymph node and one patient showed liver metastases (Table S1).

Only one patient reported dizziness during ^64^Cu-GD2 infusion, which disappeared by reducing the flow rate. No other drug-related pharmacologic effects or physiologic responses were reported.

### Image quality

**Figure 1:**
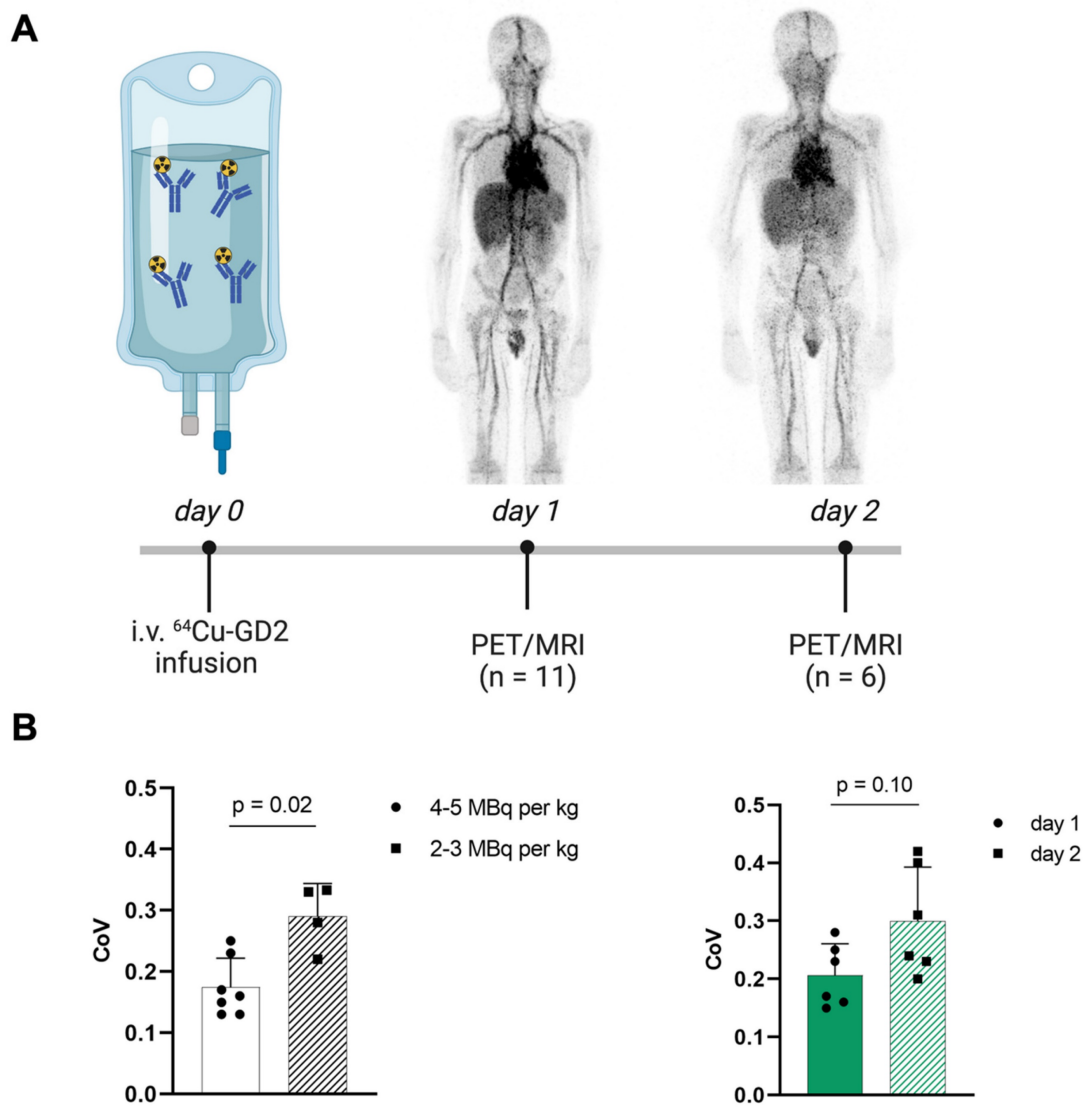
**A:** The schematic workflow of ^64^Cu-GD2 application, PET/MRI and imaging timepoints is shown. **B:** A significantly higher coefficient of variation (CoV) as a measure of poorer image quality (p < 0.05) was assessed in the four patients after injection of 2-3 MBq per kg/BW ^64^Cu-GD2 compared to the seven patients after injection of 4-5 MBq per kg/BW ^64^Cu-GD2 (left). A tendency towards lower CoV and thus improved image quality was found on day 1 compared to day 2 in six patients, which were examined at two timepoints (right). All Data are presented as the mean ± standard error and considered significant at *p* < 0.05. Partially created with BioRender.com.

Increasing injected activity from 2-3 MBq per kg/BW as initially used in the first four patients to 4-5 MBq per kg/BW in the following 7 patients improved the subjective image quality. To objectify this, the CoV was assessed (Figure 1), which showed a significantly higher CoV in the patients with lower injected ^64^Cu-GD2 activity (2-3 MBq/kg/BW: 0.3 ± 0.1 vs. 4-5 MBq/kg/BW: 0.2 ± 0.1; p = 0.02). Moreover, statistical image quality was found to decrease on day 2 in those patients with two PET/MRI imaging timepoints (n = 6), as reflected by a rise of CoV (day 1: 0.2 ± 0.1 vs. day 2: 0.3 ± 0.1; p = 0.10).

### Biodistribution and Dosimetry

**Figure 2:**
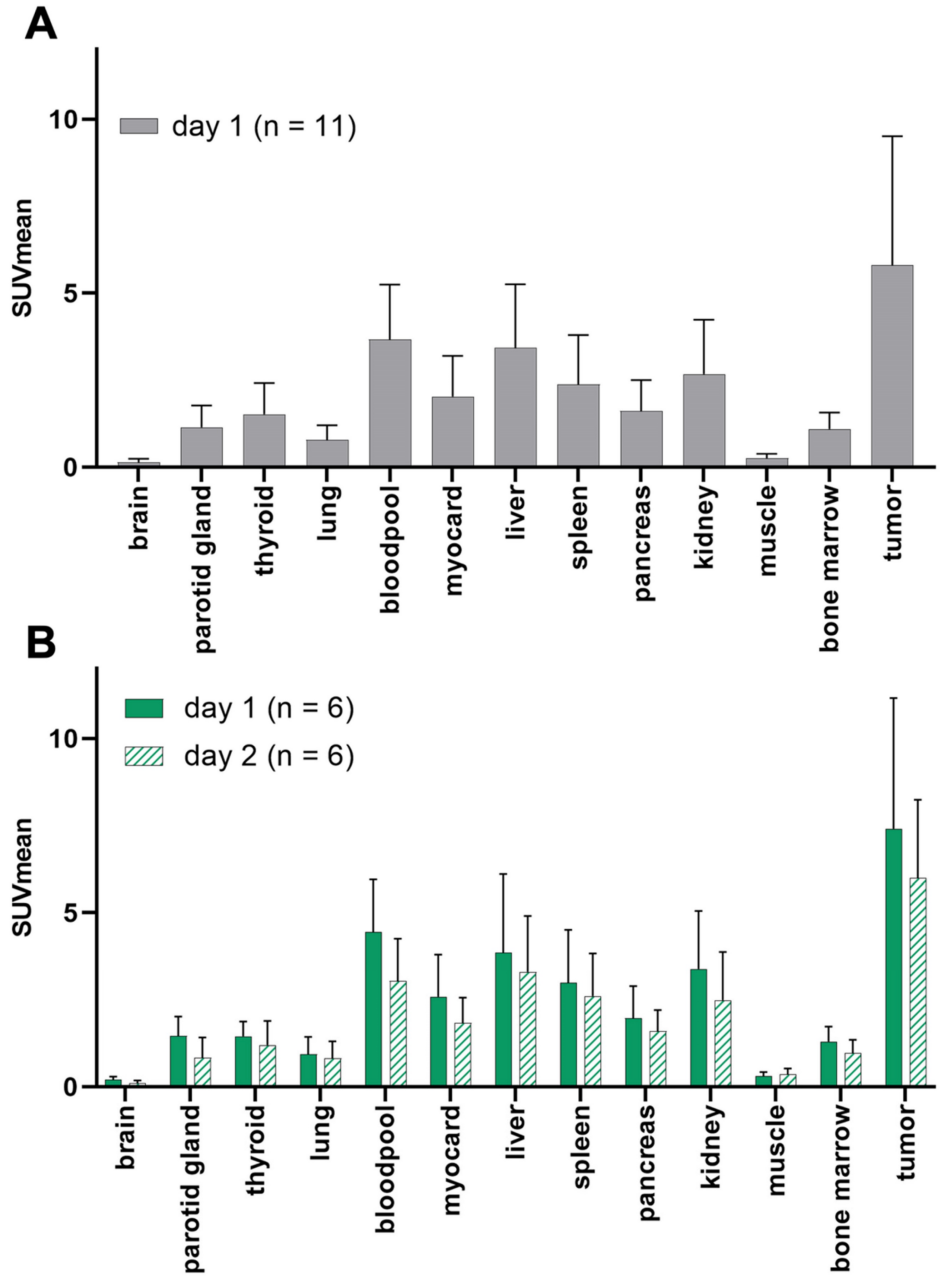
Patient based analysis of ^64^Cu-GD2 biodistribution assessed by semiquantitative measurements (SUV_mean_) in PET in all patients on day 1 (n = 11) (**A**) and in patients with two scans (n = 6) on day 1 and day 2 (**B**). **A:** Average ^64^Cu-GD2 uptake in various organs and GD2 positive tumor lesions is presented, revealing high tumor to background ratios in relevant areas such as bone marrow, lung and muscle. **B:** Decreasing ^64^Cu-GD2 uptake is found on day 2 in both organs and tumors.

In one patient, no GD2-expression could be detected in any tumor lesions. The average SUV_mean_ (5.8 ± 3.7) in representative GD2-positive lesions (n = 8) was higher than that in the lung (0.8 ± 0.4), liver (3.4 ± 1.8), muscle (0.3 ± 0.1), bone marrow (1.1 ± 0.5) and bloodpool (4.0 ± 2.1) in the total population (n = 11). TBR_mean_ was excellent in the bones (8.5), muscles (21.7), and lungs (7.9). Since ^64^CU-GD2 does not cross the blood-brain barrier, low SUV values were measured (Figure 2, Figure S1).

When comparing only the patients who had two scans (n = 6) the average ^64^Cu-GD2 uptake of the tumor lesions decreased between day 1 (SUV_mean_ 7.4 ± 3.8) and day 2 (SUV_mean_ 6.0 ± 2.3). No additional lesion was detected on day 2, when compared to day 1. Therefore, data from day 1 were used for further analyses of all patients. Normal tissue biodistribution on day 1 and day 2 is provided in Figure 2 and Figure S1.

The quantification of the absorbed dose is presented in Table 2. The highest radiation dose in normal tissue was estimated in the liver with a mean dose of 0.15 mGy/MBq (range: 0.04-0.21 mGy/MBq) and in the spleen with 0.11 mGy/MBq (range: 0.05-0.18 mGy/MBq), respectively. The estimated whole body radiation dose was 0.03 mGy/MBq (range: 0.02-0.04 mGy/MBq).

### ^64^Cu-GD2 expression in various tumor types

**Figure 3:**
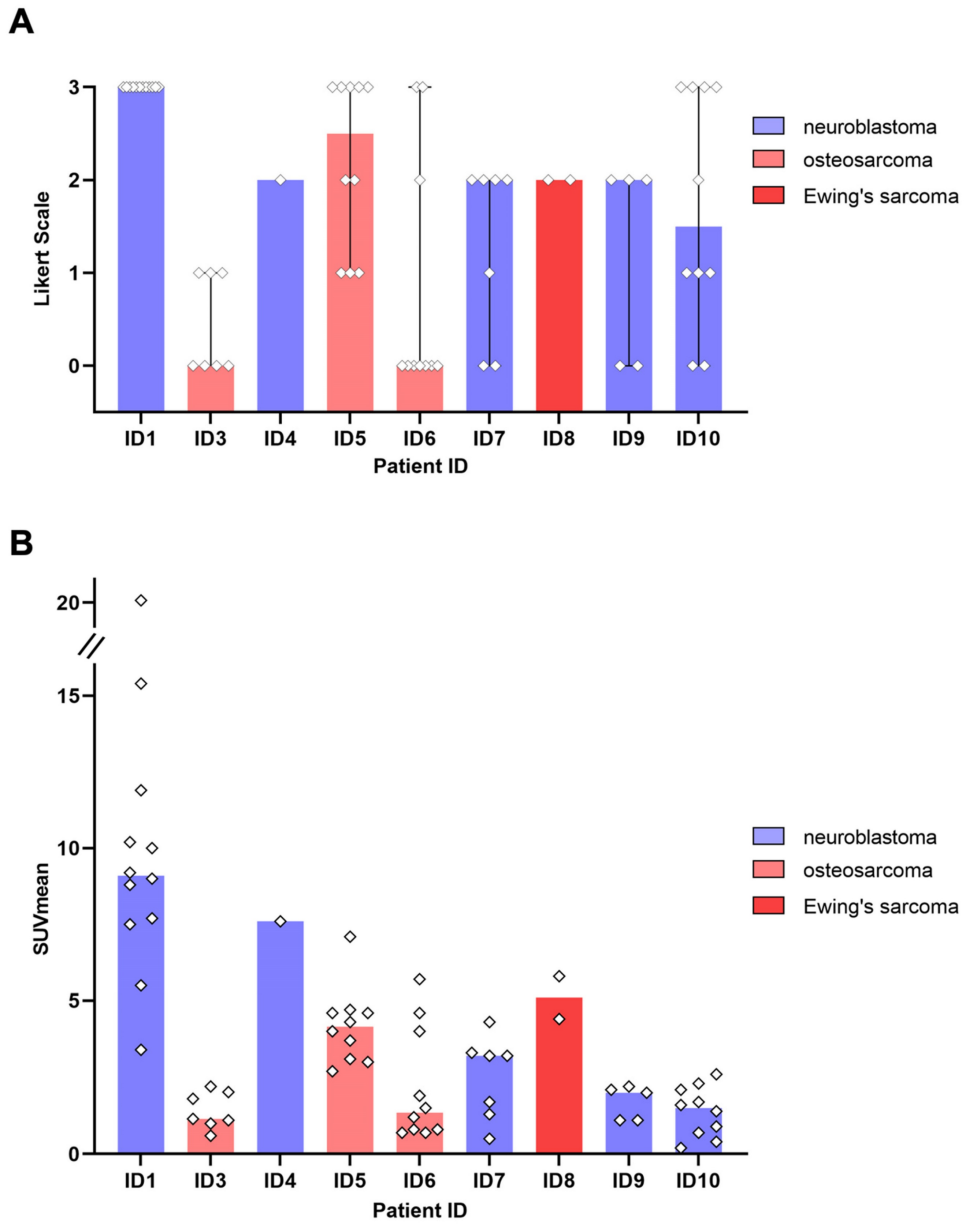
GD2 expression in tumor lesions as measured by ^64^Cu-GD2 PET/MRI in 9 patients with detectable disease. **A:** The visual rating of individual lesion uptake against background using a 4-point Likert scale is presented. **B:** Semiquantitative assessment of ^64^Cu-GD2 uptake in detectable tumor lesions (up to 10 lesions per organ) measured as SUV_mean_ is shown. The colored representation of the column indicates the respective median value.

**Figure 4:**
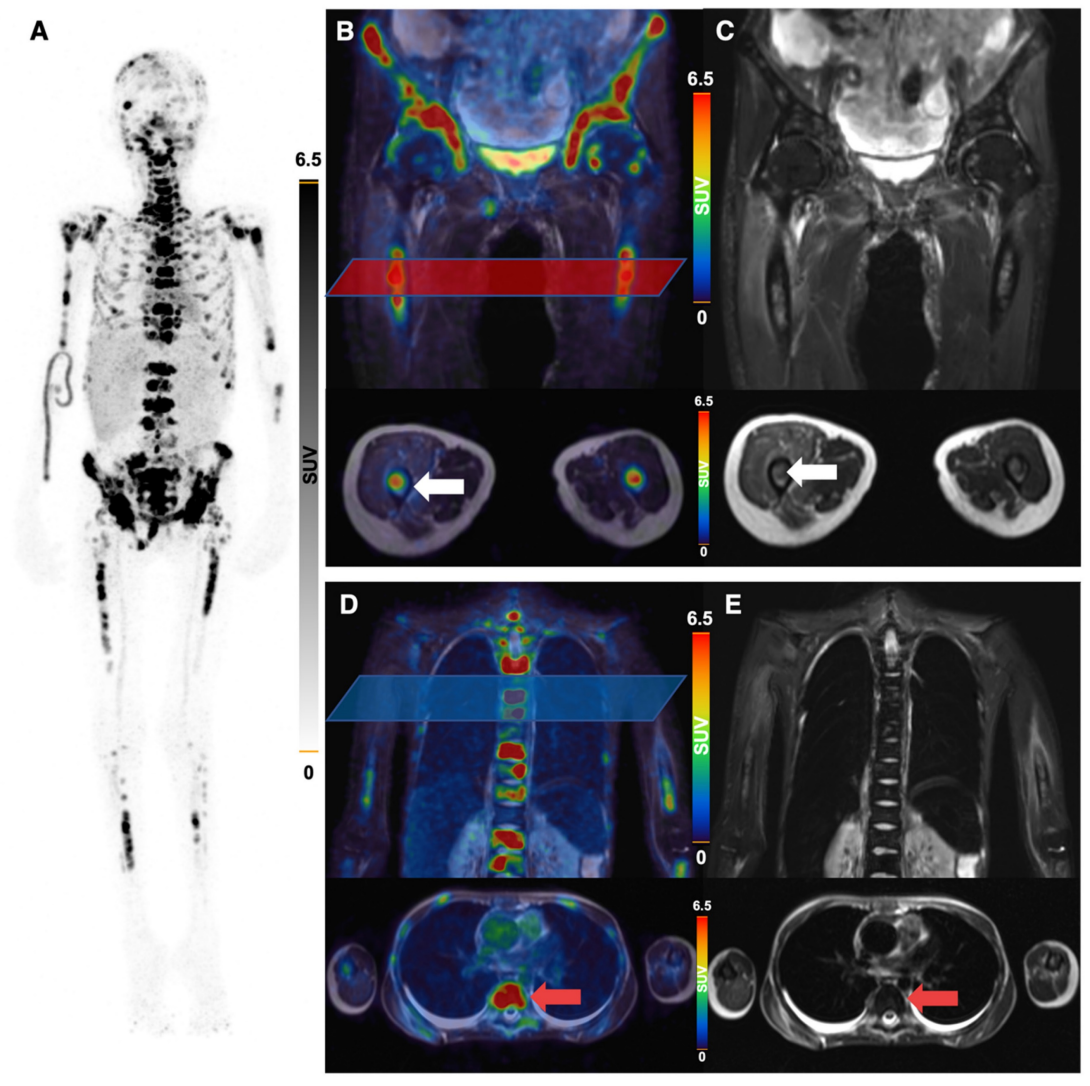
^64^Cu-GD2-PET/MRI of a 20 year old female patient (ID1) with disseminated metastatic neuroblastoma and intense GD2 expression of bone lesions. Maximum intensity projection of PET images on day 1 (**A**), coronal (upper row) and transaxial (lower row) representation of fused PET/MRI (**B, D**) and MRI (coronal T2 STIR and transaxial T2 HASTE sequence) (**C, E**) are shown. The transaxial images correspond to the area of the colored box in the coronal fused images. Multiple ^64^Cu-GD2 positive bone metastases are depicted in the skull, spine, arms and legs with a very high SUVmax up to 30.2 (**A**). ^64^Cu-GD2 positive metastases are demonstrated in both proximal femora (white arrows; **B, C**). ^64^Cu-GD2 positive bone metastases with no clear signal in MRI (red arrows; **D, E**) are shown, revealing the complimentary role of ^64^Cu-GD2 PET for tumor detection.

All 5 patients with detectable neuroblastoma lesions showed ^64^Cu-GD2 uptake in several but not all metastases. 2 of these patients presented with a high ^64^Cu-GD2 uptake (ID1, ID10), but 3 others had only a low to moderate tumor uptake (ID 4, ID7, ID9) (Figure 3A). In addition, GD2-positive tumor burden seemed to have an impact on physiological tracer distribution, as demonstrated in patient ID1, presenting with disseminated disease (Figure 4), but also quite low ^64^Cu-GD2 uptake in the liver and low bloodpool activity already on day 1, possibly indicating a tumor sink effect. The multiple bone metastases showed particularly high tumor uptake up to SUV_max_/SUV_mean_ 30.2/20.1, but only moderate ^64^Cu-GD2 uptake in the dura metastases.

**Figure 5:**
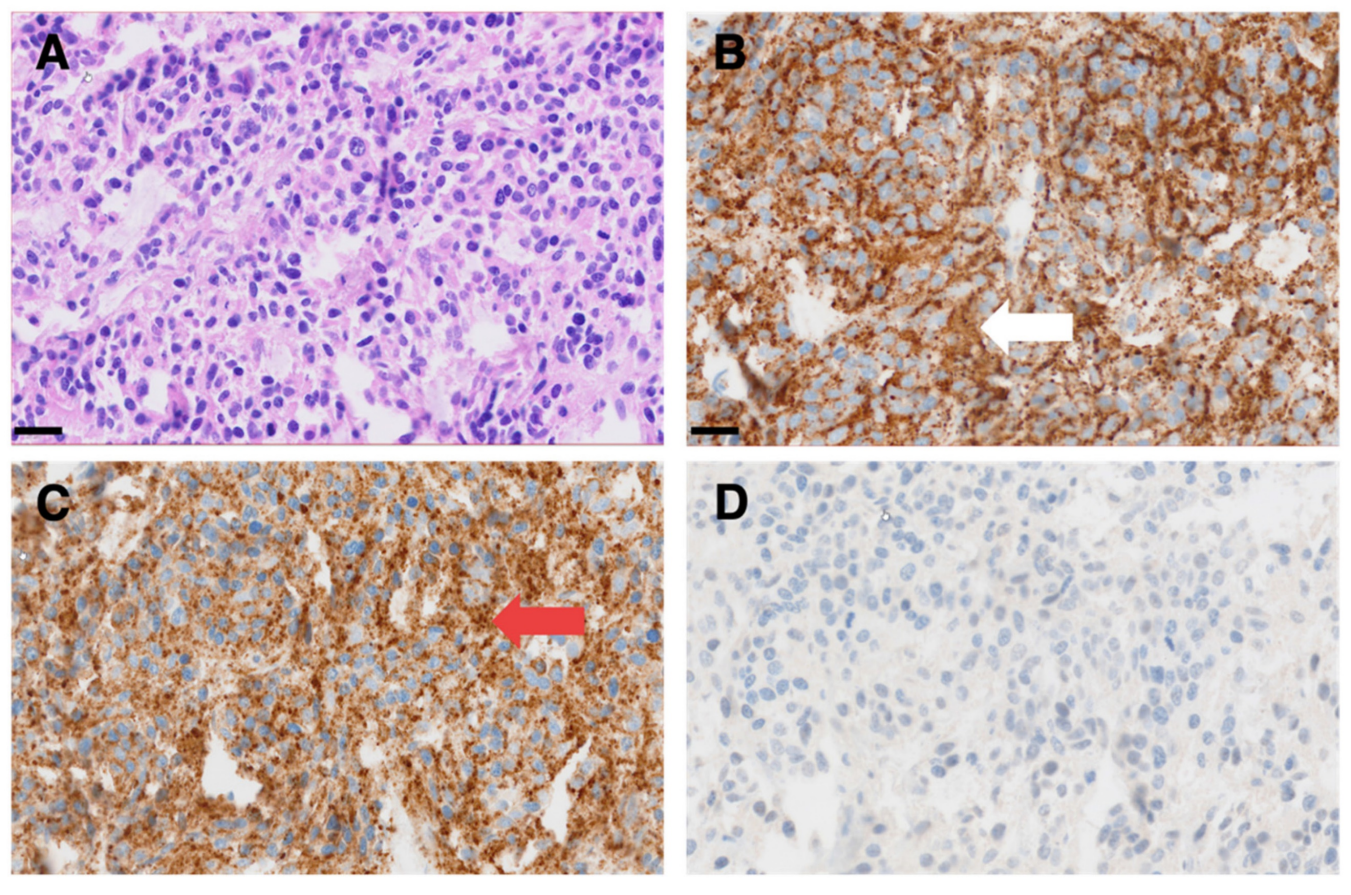
Representative micrographs of a dura metastasis specimen from patient ID1. HE staining (**A**) shows tumor cells of a poorly differentiated neuroblastoma with strong immunohistochemical positivity for synaptophysin (white arrow in **B**) and chromogranin A (red arrow in **C**), while being negative for GD2 (**D**). Scale bar = 20 µm.

Interestingly, this particular patient, was previously considered to be GD2 negative based on histological evaluation of a dura metastasis (Figure 5). The decision for PET was made due to progressive disease 8 months later and lack of further therapeutic options, demonstrating intense GD2 expression in the disseminated bone metastases thus providing a treatment target.

**Figure 6:**
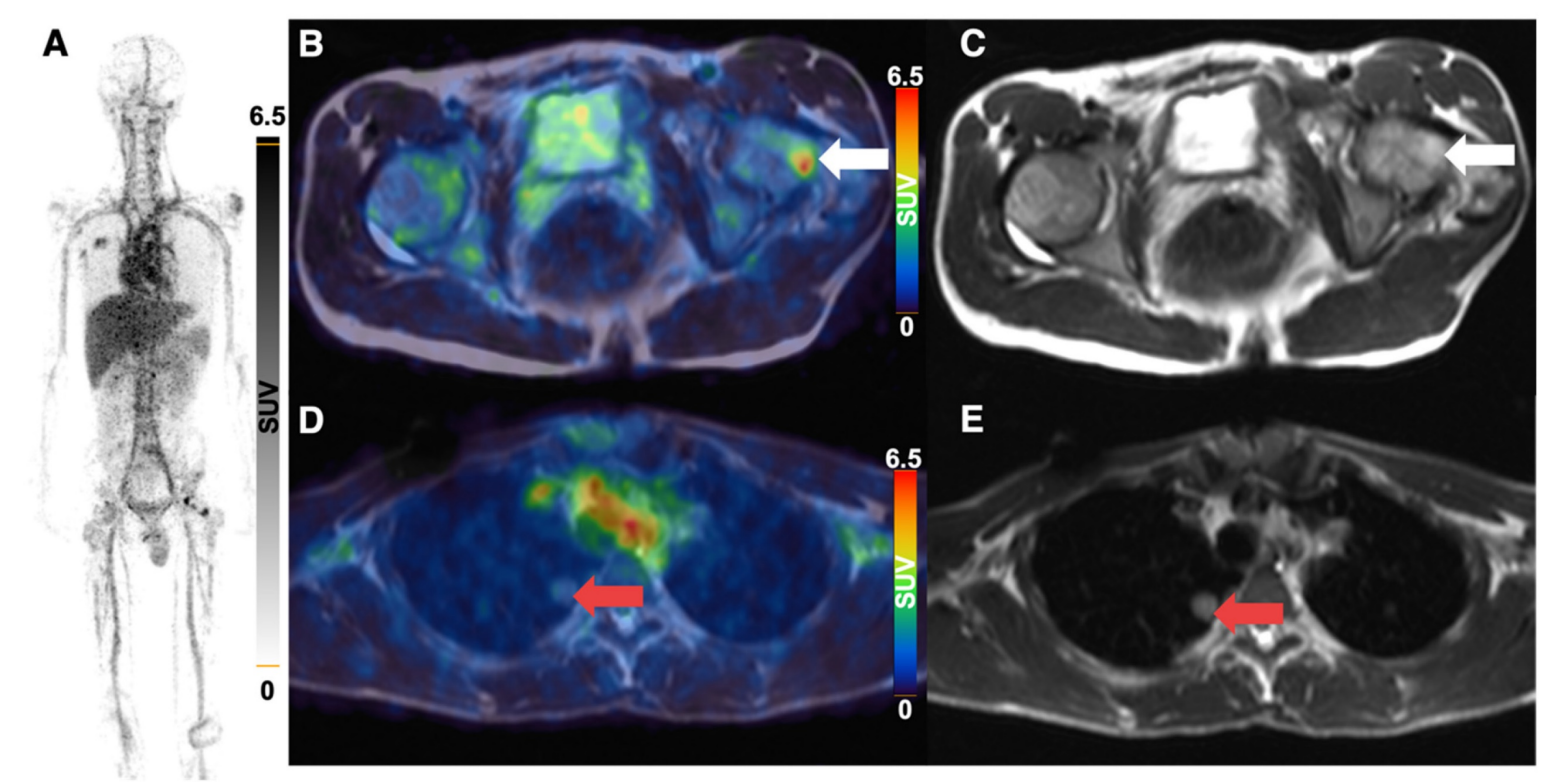
^64^Cu-GD2 PET/MRI of a 18 year old male patient with metastasized osteosarcoma (ID6). Maximum intensity projection of PET images on day 1 (**A**), transaxial representation of fused PET/MRI (**B, D**) as well as MRI (T2 HASTE sequence) (**C, E**) are shown. A ^64^Cu-GD2 positive bone metastasis (white arrow) was found in the left femur with a SUVmax of 8.5 (**B, C**). A lung metastasis (red arrow) demonstrates no significant uptake (**D, E**).

3 of 4 sarcoma patients with detectable tumor showed increased ^64^Cu-GD2 uptake in at least one tumor lesion (Figure 3A). In 2 of these patients, a heterogenous GD2 expression was detected with Likert 1 to 3 and 0 to 3. In particular, one of them (ID6) showed an intense uptake in bone lesions, but no uptake in the multiple lung and lymph node metastases of more than 3 cm transaxial diameter (Figure 6). In the remaining patient (ID3) multiple lung metastases showed no ^64^Cu-GD2 uptake. They were histologically confirmed as lacking GD2 expression after PET/MRI.

Summing up, ^64^Cu-GD2 uptake was shown to be very heterogenous in 5 of 8 patients (Figure 3).

Furthermore, we compared different MRI derived parameters of GD2 positive and negative lesions. There was no statistically significant difference in lesion size. Additionally, there was no statistically significant difference in apparent diffusion coefficient (ADC) values between GD2 positive and negative lung or bone metastases (Figure S2).

### Further therapy and imaging around ^64^Cu-GD2 PET/MRI

Treatment options were discussed by an interdisciplinary pediatric tumor board. Considering largely high GD2 expression in PET/MRI, decision was made for dinutuximab beta therapy in 5 patients (Table 3). In the other 4 cases, chemotherapy was chosen for 3 patients (ID3: gemcitabine/docetaxel; ID6: irinotecan/temozolomide; ID9: melphalan/ceritinib) while one patient received ^131^I-MIBG therapy. Progression free survival (PFS) was determined by CT, MRI or death. Various imaging modalities have been performed on these patients at different stages of their disease (Table S2). There was a trend towards longer PFS for patients receiving GD2-targeted treatment.

## Discussion

A non-invasive marker for the holistic assessment of GD2 expression and GD2 antibody tissue penetration in-vivo is highly desirable, as GD2 is considered a promising primary target for cancer immunotherapy 5. Because it's not only overexpressed in pediatric tumors such as neuroblastoma, as osteosarcoma or Ewing's sarcoma, but also in various other tumor such as small cell lung cancer, breast, glioma and soft tissue sarcoma as well as melanoma 30, a theranostic approach based on GD2 would offer a wide range of clinical applications.

This study revealed that PET imaging with ^64^Cu-GD2 enables a non-invasive visualization and quantification of GD2 expression *in-vivo* in different pediatric cancer entities such as neuroblastoma, osteosarcoma, and Ewing's sarcoma. Moreover, ^64^Cu-GD2 PET/MRI was able to reveal inter- and intraindividual heterogeneity and was associated with only low radiation burden, allowing its use in children for both treatment stratification and treatment response.

In 8 of 9 patients with evidence of tumor lesions identified in multiparametric MRI, at least one lesion showed increased ^64^Cu-GD2 uptake in PET. 4 of those 8 patients had a high ^64^Cu-GD2 uptake as defined by a Likert scale of 3 (2 neuroblastoma and 2 osteosarcoma), while 4 presented with moderate ^64^Cu-GD2 uptake (3 neuroblastoma, and 1 Ewing's sarcoma) and one patient suffering from osteosarcoma was considered as GD2 negative.

In our present analysis ^64^Cu-GD2 PET showed a high tumor to background ratio in the bone marrow, the skeletal bone, and lymph nodes which are known to be the most common metastatic sites in neuroblastoma 31, 32. Furthermore, tumor to background ratio in areas, which are typically affected by metastases in osteosarcoma and Ewing's sarcoma like muscle, bone and lung, were also high 33, 34. However, relatively high tracer uptake in the liver and spleen, as well as slow clearance of the tracer from the blood pool, may limit the assessment of GD2 expression in hepatic or splenic lesions.

As the injected activity of 2-3 MBq per kg/BW used for the first four PET/MRI scans were associated with only moderate image quality, we decided to increase the activity to 4-5 MBq per kg/BW, which resulted in a significant improvement. Both, the higher count rate for PET-scanning as well as the increased amount of radiolabeled antibody might have contributed, as the concentration remained unchanged. The total amount of CH14.18/CHO antibody administered in this study was approximately 0.08 to 0.47 mg which accounts for less than 5% of that given in clinical treatment with dinutuximab beta. Still, one patient reported dizziness during ^64^Cu-GD2 infusion. As the event disappeared by reducing the infusion flow rate, it is to be considered as an antibody-induced reaction. However, no severe adverse reactions were reported following ^64^Cu-GD2 application in this study.

Recently, we reported our experience with the radiolabeled antibody [^131^I]-GD2-ch14.18 35, using the beta- and gamma-emitting radionuclide ^131^I for Single-Photon-Emission-Computed-Tomography / X-ray-Computed-Tomography (SPECT/CT) imaging. Although [^131^I]-GD2-ch14.18 was considered to be an interesting approach for radioimmune therapy, the resulting image quality in SPECT/CT was clearly poorer than that currently achieved with ^64^Cu-GD2 PET and did not allow for (semi-) quantitative assessment of GD2 expression in tumor lesions. Moreover, whole body radiation exposure could be significantly reduced with ^64^Cu-GD2 (0.03 mGy/MBq) as compared to [^131^I]-GD2-ch14.18 (0.41 mGy/MBq). This also applies for all organ doses such as liver (0.15 mGy/MBq; 0.57 mGy/MBq), kidney (0.09 mGy/MBq; 0.57 mGy/MBq), spleen (0.15 mGy/MBq; 1.15 mGy/MBq) and lung (0.07 mGy/MBq; 0.88 mGy/MBq).

Due to the slow clearance of immunoconjugates from the blood, immunoimaging with antibodies generally calls for radionuclides with rather long half-lives such as the radiometals ^64^Cu (half-life of 12.7 h ) or ^89^Zr (half-life of 78.4 h) meaning PET imaging still comes with non-negligible radiation exposure 36, 37. However, the radiation dose was found to be significantly lower (approximately by a factor of 8 to 9) for ^64^Cu labeled tracers as compared to ^89^Zr 38-40. Remarkably, ^64^Cu-GD2 PET image quality on day 1 after injection was high despite the slow blood clearance of ^64^Cu-GD2, arguing for the use of this radioisotope instead of ^89^Zr, which otherwise would be required for even longer residence time of the tracer in blood at the cost of limited image quality in earlier images. Using new generation long-axial-field-of-view PET scanners with dramatically improved imaging quality because of higher spatial resolution and increased sensitivity has the potential to reduce the injected tracer activity 41. Thus, these new scanners will allow to decrease radiation exposure in immunoimaging, which is especially desirable in pediatric patients.

Presently GD2 is mainly used as immunotherapy target in neuroblastoma 5, 18, but other potentially GD2-positive tumors such osteosarcoma and Ewing's sarcoma are also under investigation 16, 42. Current inconsistency in GD2 immunohistochemistry staining is a serious issue that has also been recognized by the children's oncology group 43.

Therefore, in order to obtain an adequate therapy stratification but also to avoid potentially harmful and ineffective treatment, pre-therapeutic visualization of the GD2 status may be helpful. As a first proof of concept, we reported the correlation of high GD2 expression in histology with ^64^Cu-GD2 uptake in one patient with osteosarcoma 26.

For operable tumors, performing an initial ^64^Cu-GD2 scan initially could be an option to determine the expression status. If imaging results indicate GD2 positivity, targeted surgery could be performed using dinutuximab beta labeled with a fluorescence dye 44, 45. In the current patient cohort, we found that GD2 expression in neuroblastoma was heterogenous both inter- and intraindividually. This was illustrated by a patient whose dura metastasis was initially confirmed to be histologically GD2-negative, while PET performed few months later showed intense GD2-targeting in disseminated bone metastasis. This case illustrates the risk of sampling bias in metastatic tumor disease.

Taken into consideration that the primary therapy of all patients with high risk neuroblastoma comprises dinutuximab (US) or dinutuximab beta (Europe) 46, and current trials encourage to treat patients with relapsed neuroblastoma again with GD2-targeting antibodies 47, 48, our finding of GD2 negative lesions in 3 patients with GD2 positive neuroblastoma is worrisome. It challenges the current clinical practice, and strongly requires further investigation. Of note, all neuroblastoma patients in our cohort were patient with relapsed neuroblastoma that were pretreated with dinutuximab beta. Therefore, the effect of a sampling bias due to the pretreatment with dinutuximab beta remains elusive, as well as the effect of tumor evolution during initial dinutuximab treatment. Future ^64^Cu-GD2 imaging studies need to address the heterogeneity of GD2 expression in primary neuroblastoma and need to correlate the presence of GD2 negative lesions with outcome both in primary as well as relapsed neuroblastomas treated with dinutuximab or dinutuximab beta.

In addition, we showed relevant GD2 tracer uptake in cases of Ewing's sarcoma and osteosarcoma, indicating potential for GD2 directed immunotherapy. On the other hand, several lesions showed only a weak ^64^Cu-GD2 uptake. Additionally, a restriction of ^64^Cu-GD2 access might have played a role, potentially caused by tissue characteristics such as interstitial pressure or necrosis 49-52. Therefore, further investigation involving multiple imaging centers is needed to confirm the value of this approach. Our data suggest that the introduction of non-invasive GD2 imaging with PET can be considered a useful diagnostic tool for selecting personalized therapy in cancers that can express GD2. This theranostic approach has the potential to provide additional clues to GD2 expression and tissue penetration of GD2-targeting antibodies.

### Limitations

Limitations of the current study include its retrospective design and the heterogenous and small patient cohort. Furthermore, in most cases, immunohistochemical confirmation was not available for ethical reasons, as this would have required fresh tissue samples, which can only be obtained by surgical intervention.

## Conclusion

^64^Cu-GD2 PET is a very promising new imaging method for non-invasive visualization and quantification of GD2 expression in different types of cancer, especially neuroblastoma, Ewing's sarcoma and osteosarcoma. In our cohort of heavily pretreated patients, ^64^Cu-GD2 PET influenced therapy stratification. As dinutuximab beta is an already approved drug targeting GD2 and anti-GD2-CART therapies were reported to be effective, ^64^Cu-GD2 could open up a new area of image-guided therapy and thus, pave the way to GD2 theranostics.

## Supplementary Material

Supplementary figures and tables.

## Figures and Tables

**Table 1:** Patient characteristics. CTX = chemotherapy; hSCT = hematopoietic stem cell transplantation; IT = immunotherapy with dinutuximab beta; MIBG = ^131^I-MIBG therapy; RT = radiotherapy; S = surgery; NB = neuroblastoma; OS = osteosarcoma; ES = Ewing`s sarcoma

Patient ID	Age	Sex	Weight (kg)	Injected activity (MBq)	Antibody mass (mg)	Histology	Prior treatments	Tumor lesion on MRI	^64^Cu-GD2 expression on PET
1	20	F	23	114	0.23	NB	CTX, IT, MIBG, R, hSCT	+	+
2	9	F	24	110	0.22	NB	S, CTX, IT, RT	-	-
3	16	M	55	94	0.19	OS	S, Ch	+	-
4	15	F	50	200	0.40	NB	S, RT, IT, CTX	+	+
5	17	F	48	234	0.47	OS	S, CTX,	+	+
6	18	M	36	194	0.39	OS	S, CTX, RT	+	+
7	17	M	40	125	0.25	NB	CTX, hSCT, RT, MIBG, IT	+	+
8	16	M	64	163	0.33	ES	S, RT, CTX	+	+
9	4	M	16	37	0.08	NB	CTX, RT, IT	+	+
10	7	M	21	78	0.16	NB	MIBG, hSCT, CTX	+	+
11	14	M	42	202	0.40	ES	CTX, RT	-	-

**Table 2:** Estimated radiation absorbed dose in mGy/MBq (as calculated by OLINDA® software)

Site	Mean	Maximum	Minimum	n
Kidney	0.09	0.16	0.04	6
Liver	0.15	0.21	0.04	6
Lung	0.07	0.1	0.04	6
Spleen	0.11	0.18	0.05	6
Tumor	0.19	0.46	0.07	4
Whole Body	0.03	0.04	0.02	6

**Table 3:** Oncological treatment performed after ^64^Cu-GD2 PET/MRI and subsequent image-based progression free survival. For both patients without detectable tumor (ID 2 and 11) follow-up period is given. IT = immunotherapy with dinutuximab beta; MIBG = ^131^I-MIBG therapy

Patient ID	Oncological treatment performed after ^64^Cu-GD2 PET/MRI	Progression free survival (months)
1	IT	3
2	watch and wait	9
3	Gemcetabine/Docetaxel	2
4	IT	30
5	IT	4
6	Irinotecan/Temozolomide	2
7	MIBG	4
8	IT	3
9	Melphalan/Ceritinib	1
10	IT	4
11	watch and wait	6
