# Supplementary material for: A novel approach to guide GD2-targeted therapy in pediatric tumors by PET and [64Cu]Cu-NOTA-ch14.18/CHO
Source: Theranostics. 2024 Jan 20;14(3):1212–23. doi: 10.7150/thno.92481 (PMC10845206; doi:10.7150/thno.92481)

Table S1. Patient based enumeration of tumor lesions and sites detected by ^{64}Cu-GD2 PET/MRI.

Patient ID	Lung	Lymph nodes	Liver	Bone	Soft tissue
1	0	0	0	>10	2
2	0	0	0	0	0
3	7	0	0	0	0
4	0	1	0	0	0
5	>10	0	0	0	0
6	5	2	0	3	0
7	0	0	0	7	0
8	0	0	0	0	2
9	0	0	2	2	1
10	0	0	0	>10	0
11	0	0	0	0	0

Table S2. Additional functional and follow-up imaging performed (+ positivity of detected lesions, - no lesions detected, l. f. = lost to follow up, n.p. = not performed).

Patient ID	Histology	Tumor lesion on MRI	^{64}Cu-GD2 expression on PET	MIBG-SPECT/CT	FDG-PET	Follow-up imaging
1	NB	+	+	n. p.	n. p.	progress
2	NB	-	-	-	n. p.	stable
3	OS	+	-	n. p.	n. p.	progress
4	NB	+	+	+	n. p.	response
5	OS	+	+	n. p.	n. p.	progress
6	OS	+	+	n. p.	n. p.	progress
7	NB	+	+	+	n. p.	progress
8	ES	+	+	n. p.	n. p.	progress
9	NB	+	+	+	n. p.	progress
10	NB	+	+	n. p.	+	progress
11	ES	-	-	-	n. p.	l. f.

Figure S1. PET based assessment of ^{64}Cu-GD2 biodistribution by SUV_{max} analysis in all patients (n = 11) on day 1 as well as on day 1 and day 2 in patients with two imaging timepoints (n = 6).

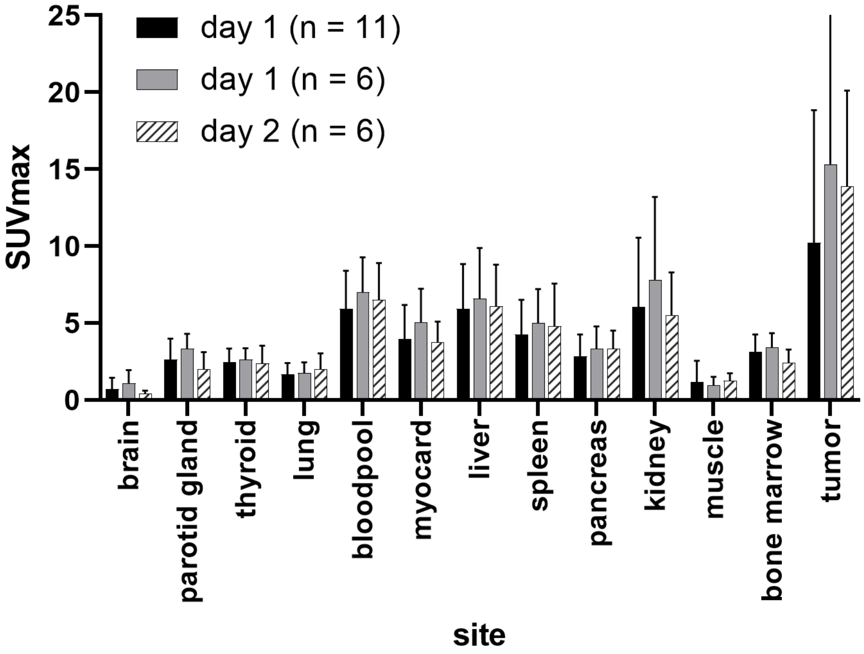

Figure S2. Comparison of ^{64}Cu-GD2 expression in PET with MRI based parameters such as tumor lesion size (long axis diameter of all lesions in cm) (**A**) and ADC values of bone metastases (**B**) or pulmonary metastases (**C**). Tumor lesions presenting with a Likert score ≥ 2 on ^{64}Cu-GD2 PET were defined as GD2 positive, whereas lesions with a Likert score < 2 were classified as GD2 negative. All Data are presented as the mean ± standard error and considered significant at $p < 0.05$.

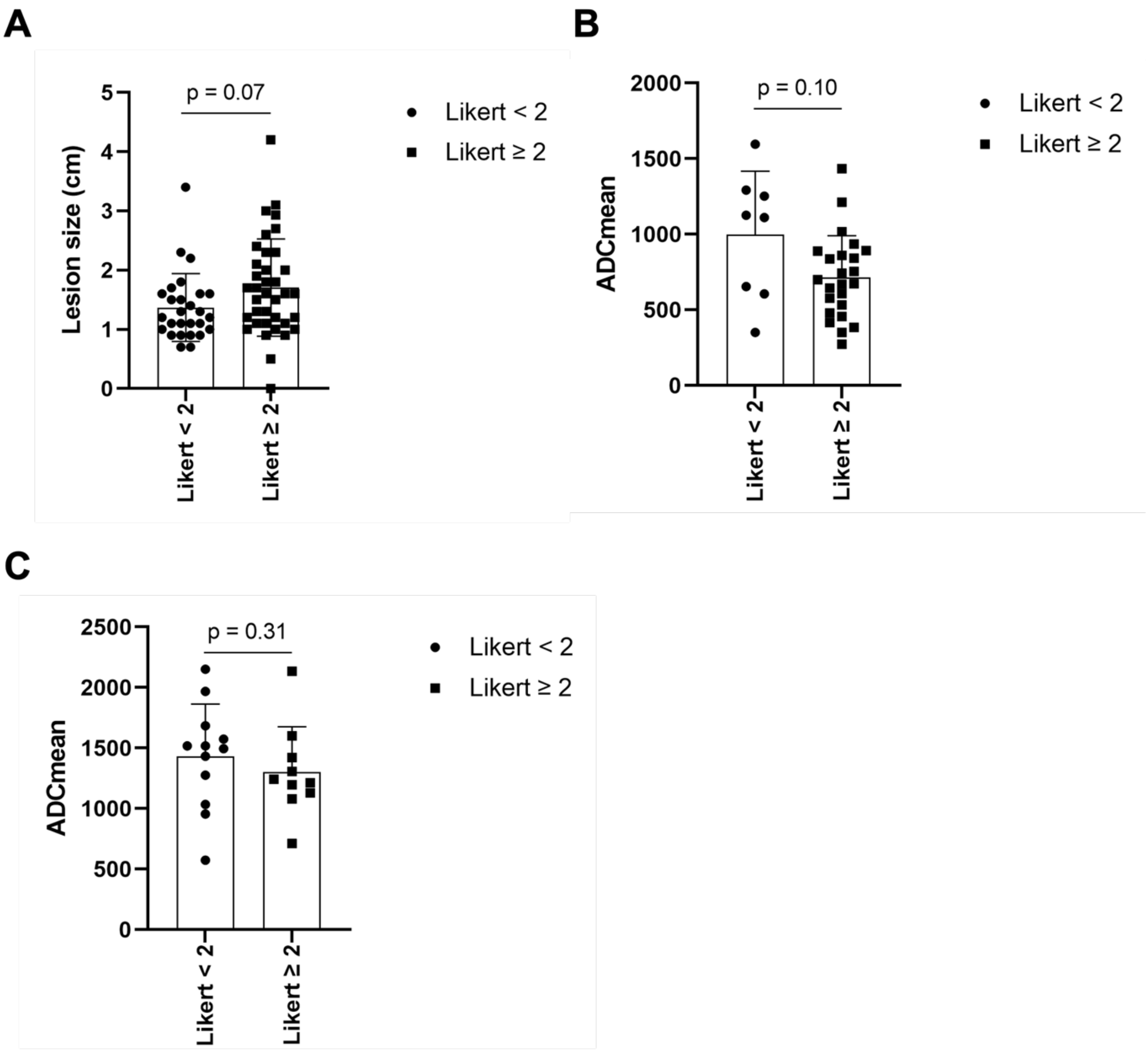

Supplement: Supplementary file 1 — Supplementary figures and tables. [file thnov14p1212s1.pdf]
